# Psychological Burden in Relapsing-Remitting Multiple Sclerosis: Sociodemographic and Clinical Determinants of Persistent Anxiety and Depression over a Six-Month Follow-Up

**DOI:** 10.3390/nursrep16020039

**Published:** 2026-01-26

**Authors:** María Lourdes Bermello López, Emilio Rubén Pego Pérez, Eva Gómez Fernández, María del Rosario Marín Arnés, Mercedes Fernández Vázquez, María Irene Núñez Hernández, Emilio Gutiérrez García

**Affiliations:** 1 Neurology and Neurosurgery of Lucus Augusti Hospital, 27004 Lugo, Spain; lubermello@hotmail.com (M.L.B.L.); buchi@hotmail.es (E.G.F.); charochili@yahoo.es (M.d.R.M.A.); mercedes.fernandez.vazquez@sergas.es (M.F.V.); irene.nunez.hernandez@sergas.es (M.I.N.H.); 2 Department of Psychiatry, Radiology, Public Health, Nursing and Medicine, University of Santiago de Compostela, 15704 Santiago de Compostela, Spain; 3 Faculty of Nursing, University of Santiago de Compostela, Avda. Xoán XXIII s/n, 15704 Santiago de Compostela, Spain; 4 Research Group CroniEduTec (Chronicity, Educational Innovation and Applied Health Technologies), Faculty of Nursing, University of Santiago de Compostela, 15704 Santiago de Compostela, Spain; 5 Research Group: GIBIOMED Group of Biomedical Research, Department of Clinical Psychology and Psychobiology, Faculty of Psychology, University of Santiago de Compostela, 15703 Santiago de Compostela, Spain; emilio.gutierrez@usc.es

**Keywords:** relapsing-remitting multiple sclerosis, health assessment, follow-up, nursing, autoimmune diseases, health status indicators, psychological distress, emotional exhaustion, anxiety, depression

## Abstract

**Background/Objectives**: Multiple sclerosis (MS) is a chronic neurological disease characterized by demyelination, inflammation, and autoimmunity, leading to progressive physical and psychological impairments. Anxiety and depression are among the most prevalent neuropsychiatric comorbidities in MS, significantly impacting patients’ quality of life (QoL). This study aimed to assess the evolution of anxiety and depression in individuals with relapsing-remitting multiple sclerosis (RRMS) over a six-month follow-up period, identify associated factors, and explore potential predictors of these psychological conditions. **Methods**: A prospective observational study was conducted with 35 RRMS patients diagnosed at the Lucus Augusti University Hospital between January 2023 and March 2025. Psychological symptoms were assessed at baseline, after 3 months, and after 6 months using the Goldberg Anxiety and Depression Scale (GADS), the Beck Depression Inventory (BDI), and the Beck Anxiety Inventory (BAI). Data were analyzed using non-parametric tests to account for the small sample size and non-normal distribution of variables. **Results**: Anxiety and depression were prevalent and persistent in the study population, with no significant changes in mean scores over time (*p* > 0.05). However, specific symptoms, such as pessimism and loss of pleasure, showed worsening trends, while sadness and guilt remained stable. Sociodemographic factors, including lower income and employment status, were significantly associated with higher anxiety and depression scores (*p* < 0.05). Additionally, clinical factors such as autoimmune comorbidities and a history of mononucleosis were linked to higher depressive symptoms. Baseline anxiety and depression scores emerged as strong predictors of future levels (*p* < 0.01), emphasizing the importance of early assessments. **Conclusions**: Anxiety and depression are prevalent and persistent in RRMS patients, with specific symptoms fluctuating over time. Sociodemographic and clinical factors play a significant role in psychological outcomes, highlighting the need for integrated care models that address both physical and psychosocial aspects of MS. Early psychological assessments and targeted interventions are critical for improving QoL and mitigating the long-term burden of mental health challenges in RRMS.

## 1. Introduction

Multiple sclerosis (MS) is a chronic, inflammatory, autoimmune, and neurodegenerative disease of the central nervous system that affects millions of individuals globally, primarily impacting the brain and spinal cord. It is one of the leading causes of neurological disability in young adults and is characterized by demyelination and axonal damage that impacts both white and gray matter in the central nervous system. Globally, MS affects approximately 2.8 million individuals, with prevalence rates varying significantly across geographical regions, ranging from 80 to 180 cases per 100,000 inhabitants in Spain to as high as 183 cases per 100,000 in Galicia [1,2,3,4,5,6]. This variability has been attributed to genetic predispositions, environmental factors, and disparities in healthcare access and diagnostic practices [7,8,9,10,11,12,13,14,15,16,17].

Its clinical manifestations are highly heterogeneous, including motor, sensory, visual, and cognitive impairments, as well as frequent neuropsychiatric symptoms such as anxiety and depression [18,19,20,21,22]. These neuropsychiatric conditions are particularly prevalent in individuals with relapsing-remitting multiple sclerosis (RRMS), the most common subtype of the disease, and significantly contribute to reduced health-related quality of life (HRQoL) [23,24,25].

Neuropsychiatric comorbidities, such as depression and anxiety, are highly prevalent in people with MS, with reported rates ranging from 25% to 65% for depression and 20% to 54% for anxiety [5,25]. These conditions not only impair emotional well-being but are also associated with faster progression of physical and cognitive disability, lower treatment adherence, and increased risk of hospitalization [26,27,28,29,30]. Moreover, depression and anxiety can manifest even before the diagnosis of MS, suggesting shared pathophysiological mechanisms between mood disorders and the disease itself [5].

Additionally, cognitive impairment affects up to 65% of people with MS, adversely affecting functions such as memory, attention, and processing speed.

Beyond pathophysiological factors, individual behaviors and characteristics play a critical role in the onset and progression of psychological symptoms in MS. For instance, smoking has been identified as a factor that exacerbates depression and fatigue while negatively impacting HRQoL [30]. Similarly, recent studies have highlighted that personality traits, such as high neuroticism, are associated with an increased risk of depression and anxiety, whereas traits like extraversion and openness may have protective effects for individuals living with MS [31].

The psychological symptoms of MS also have a profound impact on patients’ daily lives. Conditions such as anxiety and depression can lead to fatigue, sleep disturbances, and emotional distress, which together create a vicious cycle that exacerbates the overall disease burden. These symptoms hinder treatment adherence, coping mechanisms, and social well-being, emphasizing the importance of early detection and intervention [32,33,34]. Furthermore, socioeconomic factors, including lower income levels, precarious employment, and limited access to healthcare services, have been associated with higher rates and severity of these disorders, highlighting the need for equitable mental health policies and robust support systems [33].

Non-pharmacological interventions, such as mindfulness-based therapies, exercise programs, and stress reduction strategies, have demonstrated positive effects on emotional regulation, a reduction in depressive symptoms, and improvement in HRQoL among people with MS [35,36,37]. However, evidence regarding their long-term efficacy and impact on disease progression remains limited [38,39,40]. These strategies are particularly relevant given the significant socioeconomic burden associated with MS, with annual healthcare costs ranging from €10,486 to €27,217 depending on disability level, and additional non-healthcare expenses reaching up to €25,850 per individual [14,41,42,43,44,45,46]. These costs disproportionately affect low-income families, highlighting the urgent need to improve healthcare access and provide support for vulnerable populations [33].

Given the substantial impact of psychological symptoms and socioeconomic factors on disease progression and patients’ quality of life, further research is essential to better understand these interactions and inform clinical practice.

Therefore, the present study aims to evaluate the evolution of anxiety and depression in individuals diagnosed with RRMS over a six-month follow-up period in Galicia, Spain. Additionally, it seeks to identify potential associations between anxiety and depression levels and various sociodemographic, clinical, and treatment-related variables. The findings will be discussed in the context of recent evidence regarding pathophysiological mechanisms, behavioral and personality risk factors, and therapeutic interventions to address these comorbidities.

## 2. Materials and Methods

### 2.1. Study Type

This prospective observational study included 35 patients diagnosed with relapsing-remitting multiple sclerosis (RRMS) at the Lucus Augusti University Hospital between January 2023 and March 2025. Participants were assessed at three time points: baseline, three months, and six months. Psychological symptoms, including anxiety and depression, were evaluated using validated tools such as the Goldberg Anxiety and Depression Scale (GADS), the Beck Depression Inventory (BDI), and the Beck Anxiety Inventory (BAI). Additionally, health-related quality of life (HRQoL) was measured using the MSQOL-54 questionnaire to capture the multidimensional impact of RRMS on patients’ well-being. Given the small sample size and the non-normal distribution of variables, data were analyzed using non-parametric statistical tests to ensure robust results.

### 2.2. Population, Sample, and Inclusion Criteria

The target population for this study comprised individuals newly diagnosed with RRMS who were treated by physicians from the Neurology and Neurosurgery Service at HULA. Located in the city of Lugo, HULA serves a population of 332,100 residents registered with a healthcare card as of February 2017.

For the sampling strategy, all eligible patients diagnosed with RRMS during the inclusion period were recruited, covering the entire accessible population. Due to the nature of the cohort, randomization or stratification was not applicable; however, additional literature was reviewed to enhance methodological rigor and ensure alignment with best practices in observational research.

All participants were newly diagnosed with RRMS and were included at the time of diagnosis to minimize the potential confounding effects of long-term disease progression. At baseline, the mean Expanded Disability Status Scale (EDSS) score was 2.5 ± 1.0, indicating mild to moderate disability levels consistent with early-stage RRMS. None of the participants had experienced a relapse or received disease-modifying treatments (DMTs) prior to the initial assessment.

This study employed a longitudinal design over a two-year period, during which participants were included at the time of diagnosis and followed up at baseline, after three months, and after six months. The psychological impact was assessed using the GADS for depression and anxiety, the BAI for anxiety, and the BDI for depression at diagnosis and during the 3- and 6-month follow-up visits. The sample consisted of a convenience sample of individuals with RRMS, selected consecutively for the study.

Inclusion criteria required participants to be 18 years or older, residing in Galicia, and diagnosed with RRMS according to the 2017 McDonald criteria, which necessitate evidence of dissemination in space (lesions in at least two of four CNS regions: periventricular, cortical/juxtacortical, infratentorial, or spinal cord) and time (simultaneous presence of enhancing and non-enhancing lesions or new T2/enhancing lesions on follow-up magnetic resonance imaging [MRI]). Participants were also required to provide informed consent and undergo regular clinical follow-up within the framework of the Integrated Care Process for MS in Galicia.

Exclusion criteria included individuals with other MS phenotypes (e.g., primary or secondary progressive MS), psychiatric disorders diagnosed prior to the study (e.g., schizophrenia, bipolar disorder), ongoing use of psychotropic medications (e.g., antidepressants, antipsychotics, or anxiolytics), substance use disorders, or moderate to severe cognitive impairment (as determined by clinical evaluation or evidence of significant neuropsychological deficits). Additional exclusion criteria included pregnancy or lactation, refusal to participate, withdrawal of consent before completing data collection, or loss to follow-up. These criteria were chosen to minimize confounding factors that could influence psychological outcomes and to ensure that observed symptoms of anxiety and depression were primarily associated with RRMS rather than pre-existing conditions.

### 2.3. Justification and Sample Size Calculation

RRMS diagnoses represent approximately 80% of all MS cases, with MS affecting around 0.1% of the population. Based on epidemiological data, the proportion (P) was set at 0.8, reflecting that RRMS accounts for the majority of MS diagnoses. Consequently, within the Lugo healthcare area, there would be an estimated 332 individuals with MS, of which approximately 265 would have RRMS.

A 90% confidence interval was chosen to balance precision with the feasibility of conducting the study, considering its pilot nature and the operational constraints of the healthcare service. A 14% margin of error was selected to account for the expected variability in the population, given the small sample size and exploratory design of the research. The sample size was calculated using the formula:

$$\mathrm{Sample}\;\mathrm{Size}\;=\;\;\;\frac{\;\frac{z^{2}x\;P(1-P)}{e^{2}}}{1+(\frac{z^{2}\;x\;P\left(1-P\right)}{e^{2}N})}$$

The study utilized a convenience sampling method, selecting individuals who were accessible and available from the Neurology and Neurosurgery Unit and the Neurology Clinic at HULA. While this approach resulted in a high participation rate (>98%), the final sample size was limited to 35 participants due to logistical and time constraints. This reduction was attributed to the duration of the inclusion period, the availability of eligible individuals, and the operational limitations of the healthcare service during the pilot phase. The small sample size is acknowledged as a limitation of the study and is consistent with its pilot nature, which aims to explore trends rather than establish definitive conclusions.

It is important to note that the small sample size imposes limitations on the generalizability of the findings. Consequently, the results should be interpreted with caution, and future studies with larger sample sizes are necessary to validate the observed trends and strengthen the statistical power of the findings.

### 2.4. Variables

Socio-epidemiological factors: sex, age, ethnicity, education level, marital status, employment status, and annual income.

Clinical factors: presence of family history, autoimmune diseases, previous mononucleosis, pregnancy planning, tobacco and alcohol consumption, ongoing treatment, and initial symptoms.

Psychological factors: Probability of anxiety and depression (GADS), level of anxiety (BAI), and depression (BDI).

### 2.5. Instrument

#### 2.5.1. Goldberg Anxiety and Depression Scale (GADS)

GADS is a brief instrument designed to detect symptoms of anxiety and depression in both clinical and epidemiological settings. The scale comprises two subscales, one for anxiety and the other for depression, each consisting of nine items that assess both psychological and somatic symptoms. The first four items of each subscale serve as an initial screening, and if the patient scores positively on at least one of these items, the remaining five items are then administered. Each affirmative response is scored as 1 point, with total scores ranging from 0 to 9 for each subscale.

A score of 4 or higher on the anxiety subscale, or 2 or higher on the depression subscale, indicates a probable case of anxiety or depression, respectively. GADS is widely recognized for its simplicity and efficiency, requiring only a few minutes to complete, and is highly useful in both clinical practice and population-based research.

The Spanish version of the GADS was validated by Montón, Echevarría, and Campos [47] for the Spanish population. This adaptation demonstrated robust reliability and validity for detecting anxiety and depression disorders in this demographic.

#### 2.5.2. Beck Depression Inventory (BDI)

The BDI, first introduced by Beck et al. in 1996 [48] and later adapted for use in Spain, is a widely used instrument for evaluating the severity of depressive symptoms. It comprises 21 items that assess symptoms experienced over the past few weeks, with each item scored on a scale from 0 to 3, where 0 represents minimal severity and 3 represents maximum severity.

The total score ranges from 0 to 63, with a cutoff of 14 points indicating clinically significant depression. The administration of the scale typically takes about 10 min. Based on the overall score, depressive symptoms are categorized into four groups: no depression (0–13 points; group 1), mild depression (14–19 points; group 2), moderate depression (20–28 points, group 3), and severe depression (29–63 points, group 4) [48,49].

#### 2.5.3. Beck Anxiety Inventory (BAI)

The BAI, developed by Beck et al., is a self-report instrument designed to measure the severity of anxiety symptoms. It consists of 21 items that evaluate physical and cognitive symptoms experienced over the past week, with each item scored on a four-point scale ranging from 0 (“not at all”) to 3 (“severely”). The total score ranges from 0 to 63, and anxiety severity is categorized into four groups: minimal anxiety (0–7, group 1), mild anxiety (8–15, group 2), moderate anxiety (16–25, group 3), and severe anxiety (26–63, group 4). The BAI is widely recognized for its reliability and validity, particularly for its ability to distinguish anxiety symptoms from depression by focusing on somatic indicators. The inventory requires approximately 5–10 min to complete, making it practical for clinical and research use. The Spanish version of the BAI has been validated, demonstrating strong psychometric properties, including reliability, factor validity, and discriminant validity, in Spanish-speaking populations [50].

### 2.6. Data Collection

The data for this study were collected exclusively by the principal investigator and were accessible only to them and the collaborative research team. No modifications to the data were permitted by individuals outside the research team. Data collection was conducted at three defined time points: at baseline (initial diagnosis in the Neurology Unit or Clinic), after three months, and after six months (both in the Neurology Nursing Clinic).

To evaluate the psychological impact, the GADS, BDI, and BAI were administered. These instruments were applied by trained nursing staff, and the data were recorded in a collection notebook specifically designed for this study. All information was anonymized and reviewed to ensure its accuracy and consistency.

To maintain methodological rigor and standardization during data collection, all personnel involved underwent specialized training at the Faculty of Nursing and the Faculty of Psychology at the University of Santiago de Compostela. The training provided comprehensive instruction on the use, scoring, and interpretation of the instruments. Sessions combined theoretical knowledge with practical exercises, including trial applications, to ensure consistency in administration and data recording across all team members.

### 2.7. Handling of Missing Data

There were no instances of missing data in this study, as all participants successfully completed the entire data collection process. This outcome was ensured through the application of strict data monitoring protocols and consistent, effective communication with participants throughout the duration of the study.

### 2.8. Data Confidentiality and Ethical Considerations

This study was approved by the Santiago–Lugo Research Ethics Committee (Registration Code: 2022_388 on 15 December 2022). It was carried out in full compliance with the ethical principles established in the World Medical Association’s Declaration of Helsinki (2024) and adhered to current Spanish legislation, including Organic Law 3/2018 on Personal Data Protection and Guarantee of Digital Rights, Law 41/2002 on Patient Autonomy, and Law 3/2005 on access to electronic medical records.

To safeguard participant privacy, clinical data were coded and dissociated, ensuring that no identifiable information was included in the database. Only the principal investigator had access to the key linking the data to individual participants, and all information was handled in accordance with established protocols to ensure anonymity. Upon completion of the study, the data will either be destroyed or retained in an anonymized format, as specified in the informed consent provided by participants. The HULA is designated as the center responsible for data processing.

### 2.9. Data Analysis

A descriptive analysis was conducted to summarize the data. For quantitative variables, measures of central tendency, such as the mean (M), and measures of dispersion, such as the standard deviation (SD), were calculated. For qualitative variables, absolute frequencies and percentages were reported. The chi-square test was employed to evaluate the relationship between sociodemographic and clinical variables.

The normality of the data was assessed using the Shapiro–Wilk test. However, given that the “severe” category is defined as scores ranging from 29 to 63 points, we deemed it appropriate to use non-parametric tests for the analyses. Specifically, a Kruskal–Wallis test was employed to compare differences between groups at baseline, and post hoc pairwise comparisons were conducted using the Mann–Whitney U test with Bonferroni correction to control for Type I error. To account for the longitudinal nature of the study, a Friedman test was applied to assess differences across the three time points (baseline, three months, and six months) for related samples. Additionally, Wilcoxon signed-rank tests were used to compare paired differences between two time points. Given the limited sample size and the exploratory nature of the study, the results of post hoc analyses should be interpreted with caution, as they aim to identify potential trends rather than establish definitive conclusions. This approach was chosen to address the study design, the sample size, and the characteristics of the data while ensuring the validity of the statistical assumptions. All analyses were conducted using continuous variables (e.g., BDI, BAI, and GADS scores) to reflect the nature of the data and avoid the creation of ordinal groups (Groups 1–4). Assumptions for each statistical test were carefully verified, and corrections (e.g., Bonferroni for multiple comparisons) were applied as needed to ensure robust and valid results.

All statistical analyses were performed using PASW statistical software (version 23.0; SPSS Inc., Chicago, IL, USA), with a bilateral significance level set at *p* < 0.05.

GenAI (OpenAI. (2026). gpt-4o [Artificial intelligence language model]. from https://openai.com (accessed on 1 January 2026)) was used for superficial text editing, such as grammar corrections and language refinement, without influencing the scientific content, data analysis, or interpretation of results. Its use complies fully with the journal’s policies on AI usage.

## 3. Results

### 3.1. Sociodemographic Characteristics

The sample consisted of 35 participants with a mean age of 38.29 ± 10.38 years (range: 19–59 years). Of these, 57.1% were women, and 42.9% were men. Regarding age distribution, 20% of participants were aged 28 years or younger, 34.3% were between 29 and 38 years old, and 45.7% were over 39 years old. All participants were of Caucasian ethnicity. Concerning employment status, 42.9% were employed, 31.4% were self-employed, 22.9% were students, and 2.9% were retirees. More than half of the participants were married (54.3%), while 40% were single, and 5.7% were cohabiting.

In terms of educational level, 40% held a university degree, 22.9% had completed secondary education, 14.3% held advanced vocational training qualifications, 11.4% had intermediate vocational training, and 11.4% had a high school diploma. The average annual income was €17,062.86 ± €14,842.21, ranging from €0 to €60,000. Among the participants, 40% earned less than €12,450 annually, while 31.4% had an income between €20,200 and €35,200.

Regarding personal medical history, 20% reported relevant family medical history, 5.7% had a history of mononucleosis, and 17.1% had autoimmune diseases. Additionally, 22.9% were smokers, 8.6% consumed alcohol, and only 2.9% reported cannabis use. In terms of initial symptoms, 31.4% reported hypoesthesia, 20% reported visual disturbances or paresthesia, 17.1% reported diplopia, and 11.4% reported muscle weakness.

All participants underwent MRI, and the treatments received were varied. The most frequently administered treatments included ocrelizumab (20%), cladribine (14.3%), and natalizumab (14.3%). Other drugs, such as alemtuzumab, ponesimod, or ublituximab, were used in smaller proportions (2.9% each).

**Figure 1:**
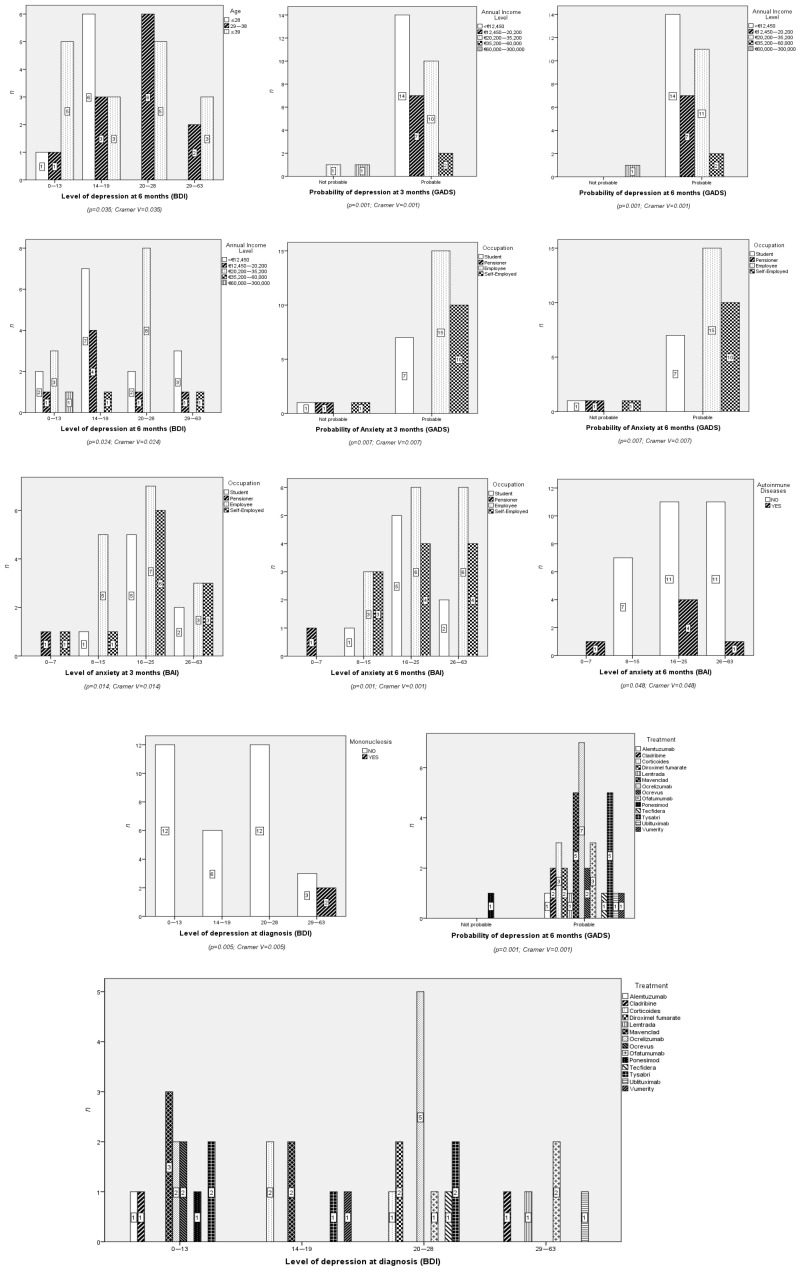
Significant results for chi-square test between sociodemographic and clinical variables.

In the depression scales, significant associations were observed with several sociodemographic variables. On the GADS, participants with lower incomes (<€12,450) were predominantly classified as “Probable” cases at both 3 and 6 months. On the Beck scale, the highest categories (3 and 4) were associated with lower incomes and a history of previous mononucleosis. While the study reports the distribution of disease-modifying treatments (DMTs) used by participants (e.g., cladribine, natalizumab, and ocrelizumab), the small sample size in each drug subgroup limits the ability to draw definitive conclusions about their specific effects on anxiety and depression scores. These observations should be interpreted as exploratory and hypothesis-generating rather than as evidence of causal relationships. Future studies with larger sample sizes are needed to validate these findings and assess the potential impact of specific DMTs on psychological outcomes. (Figure 1).

### 3.2. Psychological Impact

In the anxiety scales, most participants were classified in the highest categories across all three time points. On the GADS, 91.4% of participants were classified as “Probable” at baseline, 3 months, and 6 months, with no significant changes in distribution. On the Beck scale, an increasing proportion of participants were classified in the highest categories (3 and 4) at 3 and 6 months, particularly among students and employed participants. In contrast, retirees and self-employed individuals were primarily distributed in the lower categories. Additionally, participants with autoimmune diseases tended to fall into the highest categories (3 and 4) on this scale.

**Figure 2:**
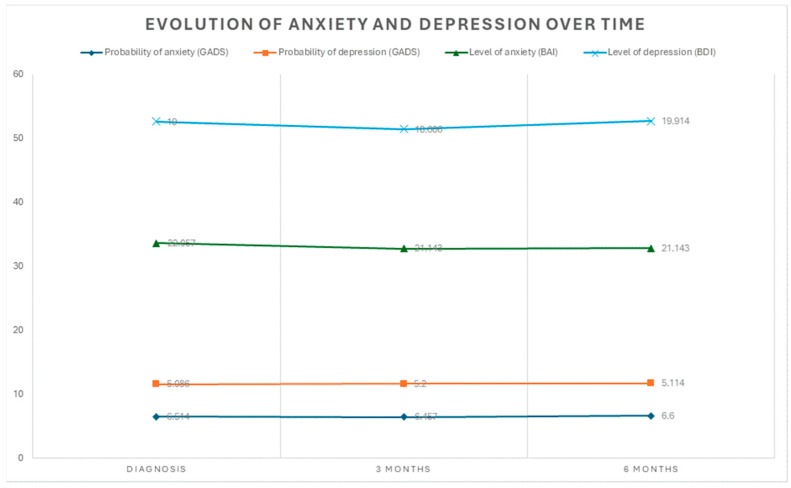
Evolution of probability and levels of anxiety and depression.

The descriptive statistics provide the means and standard deviations of the probabilities and severity levels of anxiety and depression across the three evaluation points: diagnosis, 3 months, and 6 months. Regarding the probability of anxiety (GADS), the initial mean was 6.514 (SD = 2.2278), which remained relatively stable at 3 months (M = 6.457; SD = 2.0050) and 6 months (M = 6.600; SD = 1.8974). Similarly, the probability of depression (GADS) showed an initial mean of 5.086 (SD = 2.5710), with slight variations at 3 months (M = 5.200; SD = 2.2596) and 6 months (M = 5.114; SD = 2.1662). On the other hand, anxiety levels (BAI) had an initial mean of 22.057 (SD = 10.2640), with a slight decrease at 3 months (M = 21.143; SD = 8.9644), which remained consistent at 6 months (M = 21.143; SD = 8.5307). Finally, depression levels (BDI) showed an initial mean of 19.000 (SD = 9.0326), which slightly decreased at 3 months (M = 18.686; SD = 7.8207) but increased again at 6 months (M = 19.914; SD = 8.4344). (Figure 2).

The analysis of individual BDI symptoms over time revealed significant variations in their frequency across the three time points (diagnosis, 3 months, and 6 months), as shown in Table 1. For example, the symptom of pessimism showed a temporary decrease at 3 months (57.1% of patients with a score of 1 vs. 42.9% at diagnosis) but increased again at 6 months (31.4% of patients with a score of 2 vs. 20.0% at 3 months). Symptoms such as sadness remained stable over time, with most patients consistently reporting a score of 1 (65.7% at diagnosis and 3 months, 62.9% at 6 months). Conversely, symptoms like failure and loss of pleasure exhibited a trend toward higher scores (2 and 3) over time, indicating a worsening in these specific aspects of depression.

For the anxiety symptoms analyzed using the BAI, Table S2 shows that some symptoms, such as tingling or numbness, displayed a progressive increase in higher scores (2 and 3), rising from 54.3% at diagnosis to 57.2% at 6 months. In contrast, symptoms such as fear of the worst happening decreased in severity over time, with higher scores (3) dropping from 34.3% at diagnosis to 28.6% at 6 months. Other symptoms, such as inability to relax and nervousness, persisted over time, with high scores (3) reported by 28.6% and 45.7% of patients, respectively, at diagnosis, and only slight decreases at 6 months (31.4% and 28.6%, respectively).

Guilt and feelings of punishment remained relatively stable, with a higher proportion of patients reporting low scores (0 and 1) across all three assessments. Regarding more severe symptoms, such as suicidal thoughts or ideation, most patients (82.9% at diagnosis and 77.1% at 6 months) did not report this symptom (score 0), although a small percentage (14.3% to 22.9%) reported scores of 1, highlighting the importance of continuous monitoring. (Table S1).

Some symptoms, such as tingling or numbness, show a progressive increase in higher scores (2 and 3), rising from 54.3% at diagnosis to 57.2% at 6 months, indicating a slight intensification of the symptom in some patients. On the other hand, symptoms such as a feeling of heat and dizziness or lightheadedness remained relatively stable, with a high proportion of patients reporting low scores (0 and 1) across all three time points.

For more severe symptoms, such as fear of the worst happening, there was a decrease in higher scores (3), which dropped from 34.3% at diagnosis to 28.6% at 6 months, potentially indicating an improvement in this specific aspect of anxiety. However, other symptoms, such as inability to relax and nervousness, showed greater persistence, with high scores (3) reported by 28.6% and 45.7% of patients, respectively, at diagnosis, and only a slight decrease at 6 months (31.4% and 28.6%, respectively). (Table S2)

Conversely, symptoms such as pallor and facial flushing were reported less frequently, with most patients scoring 0, suggesting these symptoms were less prevalent in this sample. Finally, fear of dying showed a trend toward a decrease in higher scores (3), dropping from 17.1% at diagnosis to 2.9% at 6 months, which could reflect an improvement in coping with this symptom.

#### 3.2.1. Depression

When comparing the probability of depression scores (GADS) with those obtained at 3 months, no statistically significant differences were found using the Wilcoxon test (Z = −0.470, *p* = 0.639). Similarly, the comparison between the initial scores and those at 6 months also showed no significant differences (Z = −0.099, *p* = 0.921). Finally, when comparing depression scores (GADS) between 3 and 6 months, no statistically significant differences were observed (Z = −0.041, *p* = 0.967).

The analysis of BDI scores across the three time points (baseline, 3 months, and 6 months) was conducted using the Friedman test, which did not reveal statistically significant differences between the measurements (χ^2^(2) = 3.866, *p* = 0.145). However, given the exploratory nature of the study and to identify potential trends in BDI scores over time, post hoc pairwise comparisons were conducted using the Wilcoxon signed-rank test. Significant differences were found between baseline and 3 months (Z = −5.164, *p* < 0.001), baseline and 6 months (Z = −5.162, *p* < 0.001), and 3 months and 6 months (Z = −5.089, *p* < 0.001). These results suggest that, although the global Friedman test did not reach statistical significance, meaningful differences in BDI scores were observed between specific time points, indicating potential changes that warrant further exploration.

The Kruskal–Wallis test was conducted to evaluate differences in BDI scores between four severity groups (asymptomatic, mild, moderate, and severe) at three time points (baseline, 3 months, and 6 months). The test revealed significant differences in BDI scores between the groups at baseline (χ^2^(3) = 31.110, *p* < 0.001), 3 months (χ^2^(3) = 26.392, *p* < 0.001), and 6 months (χ^2^(3) = 22.867, *p* < 0.001).

To identify which specific groups differed from each other, pairwise comparisons were conducted using the Mann–Whitney U test with Bonferroni correction (*p* < 0.0083). Pairwise comparisons using the Mann–Whitney U test were conducted to identify differences in BDI scores between depression severity levels at 3 and 6 months. Significant differences were observed between participants with minimal depression (BDI = 0–13) and mild depression (BDI = 14–19) at 3 months (U = 0, Z = −3.391, *p* = 0.001) and 6 months (U = 3, Z = −3.097, *p* = 0.002). Similarly, participants with minimal depression (BDI = 0–13) had significantly lower BDI scores compared to those with moderate depression (BDI = 20–28) at 3 months (U = 0, Z = −4.163, *p* < 0.001) and 6 months (U = 5.5, Z = −3.852, *p* < 0.001). Furthermore, significant differences were found between participants with minimal depression (BDI = 0–13) and severe depression (BDI = 29–63) at both 3 months (U = 0, Z = −3.174, *p* = 0.002) and 6 months (U = 0, Z = −3.166, *p* = 0.002). No significant differences were identified between participants with mild depression (BDI = 14–19) and moderate depression (BDI = 20–28) at 3 months or 6 months. Similarly, no significant differences were observed between participants with mild depression (BDI = 14–19) and severe depression (BDI = 29–63) at 3 months or 6 months. However, participants with moderate depression (BDI = 20–28) had significantly lower BDI scores than those with severe depression (BDI = 29–63) at 3 months (U = 9, Z = −2.218, *p* = 0.027), with a marginally significant difference observed at 6 months (U = 11, Z = −2.010, *p* = 0.044).

Pairwise comparisons were conducted to evaluate differences in BDI scores between depression severity levels at 3 and 6 months. Significant differences were observed between participants with minimal depression (BDI = 0–13) at 3 months and those with mild depression (BDI = 14–19) at 6 months (U = 9, Z = −3.085, *p* = 0.002), moderate depression (BDI = 20–28) at 6 months (U = 3, Z = −3.636, *p* < 0.001), and severe depression (BDI = 29–63) at 6 months (U = 0, Z = −2.505, *p* = 0.012). Participants with mild depression (BDI = 14–19) at 3 months also showed significant differences compared to those with moderate depression (BDI = 20–28) at 6 months (U = 31, Z = −2.162, *p* = 0.031) and severe depression (BDI = 29–63) at 6 months (U = 1, Z = −2.419, *p* = 0.016). No significant differences were found between participants with moderate depression (BDI = 20–28) at 3 months and those with severe depression (BDI = 29–63) at 6 months (U = 7, Z = −1.591, *p* = 0.112).

#### 3.2.2. Anxiety

A non-parametric Wilcoxon signed-rank test was conducted to evaluate changes in the scores over time. When comparing probability anxiety scores (GADS) at 3 months with baseline scores, no statistically significant differences were observed (Z = −0.273, *p* = 0.785). Similarly, the comparison of probability anxiety scores (GADS) at 6 months with baseline scores also revealed no significant differences (Z = −0.493, *p* = 0.622).

These findings suggest that there were no significant changes in the probability of anxiety between baseline and the evaluations conducted at 3 and 6 months, indicating stability in the scores throughout the follow-up period.

The analysis of BAI scores across the three time points (baseline, 3 months, and 6 months) was conducted using the Friedman test, which did not reveal statistically significant differences between the measurements (χ^2^ (2) = 0.938, *p* = 0.625)). However, given the exploratory nature of the study and the aim to detect potential patterns of change, post hoc pairwise comparisons were conducted using the Wilcoxon signed-rank test. Significant differences were found between baseline and 3 months (Z = −5.162, *p* < 0.001), baseline and 6 months (Z = −5.162, *p* < 0.001), and 3 months and 6 months (Z = −5.162, *p* < 0.001). These results suggest that, although the global Friedman test did not reach statistical significance, meaningful differences in BAI scores were observed between specific time points, which may indicate relevant patterns of change that warrant further investigation.

To identify which specific groups differed from each other, pairwise comparisons were conducted using the Mann–Whitney U test with Bonferroni correction (*p* < 0.0083). Pairwise comparisons using the Mann–Whitney U test were conducted to identify differences in BAI scores between anxiety severity levels at 3 and 6 months. No significant differences were observed between participants with asymptomatic anxiety (BAI = 0–7) and mild anxiety (BAI = 8–15) at 3 months and 6 months. Participants with asymptomatic anxiety (BAI = 0–7) had significantly lower BAI scores compared to those with moderate anxiety (BAI = 16–25) at 3 months (U = 0.500, Z = −2.638, *p* = 0.008) and 6 months (U = 0.001, Z = −2.692, *p* = 0.007). Furthermore, significant differences were found between participants with asymptomatic anxiety (BAI = 0–7) and severe anxiety (BAI = 26–63) at both 3 months (U = 0.000, Z = −2.622, *p* = 0.009) and 6 months (U = 0.000, Z = −2.610, *p* = 0.009).

Additionally, significant differences were observed between participants with mild anxiety (BAI = 8–15) and moderate anxiety (BAI = 16–25) at 3 months (U = 2.000, Z = −2.845, *p* = 0.004) and at 6 months (U = 8.000, Z = −2.274, *p* = 0.023). Significant differences were also found between participants with mild anxiety (BAI = 8–15) and severe anxiety (BAI = 26–63) at 3 months (U = 0.000, Z = −2.932, *p* = 0.003) and 6 months (U = 1.000, Z = −2.799, *p* = 0.005).

Finally, participants with moderate anxiety (BAI = 16–25) had significantly lower BAI scores than those with severe anxiety (BAI = 26–63) at 3 months (U = 19.500, Z = −3.561, *p* < 0.001) and 6 months (U = 29.500, Z = −3.098, *p* = 0.002).

Pairwise comparisons evaluated differences in BAI scores between anxiety severity levels at 3 and 6 months. No significant differences were observed between participants with asymptomatic anxiety (BAI = 0–7) and mild anxiety (BAI = 8–15) at 6 months. Participants with asymptomatic anxiety (BAI = 0–7) had significantly lower BAI scores compared to those with moderate anxiety (BAI = 16–25) at 6 months (U = 1.000, Z = −2.147, *p* = 0.032). Furthermore, significant differences were found between participants with asymptomatic anxiety (BAI = 0–7) and severe anxiety (BAI = 26–63) at 6 months (U = 0.000, Z = −2.095, *p* = 0.036).

Significant differences were also observed between participants with mild anxiety (BAI = 8–15) and moderate anxiety (BAI = 16–25) at 6 months (U = 9.500, Z = −3.243, *p* = 0.001). Similarly, participants with mild anxiety (BAI = 8–15) had significantly lower BAI scores compared to those with severe anxiety (BAI = 26–63) at 6 months (U = 0.000, Z = −3.243, *p* = 0.001).

Finally, participants with moderate anxiety (BAI = 16–25) had significantly lower BAI scores than those with severe anxiety (BAI = 26–63) at 6 months (U = 23.500, Z = −2.706, *p* = 0.007).

#### 3.2.3. Depression and Anxiety Relation

Spearman’s rank correlation coefficients showed significant positive correlations (*p* < 0.01) between anxiety and depression at diagnosis, 3 months, and 6 months. (Table 1).

The Wilcoxon signed-rank test revealed statistically significant differences between BDI and BAI scores at all three time points. At diagnosis, all 35 participants had higher BDI scores compared to BAI scores (Z = −5.163, *p* < 0.001), with no ties or positive ranks observed. At 3 months, the trend persisted, with all participants showing higher BDI scores than BAI scores (Z = −5.161, *p* < 0.001), and no ties or positive ranks were found. Similarly, at 6 months, all participants continued to have higher BDI scores than BAI scores (Z = −5.162, *p* < 0.001), with no ties or positive ranks observed. These results consistently demonstrate that BDI scores were significantly higher than BAI scores across all time points.

The Kruskal–Wallis test was used to compare BAI scores across the four BDI severity groups (1: minimal depression [0–13], 2: mild depression [14–19], 3: moderate depression [20–28], and 4: severe depression [29–63]) at diagnosis, 3 months, and 6 months. Significant differences in BAI scores were found between the groups at all three time points. At diagnosis, there were significant differences (χ^2^ (3) = 16.976, *p* = 0.001), with higher BDI severity levels corresponding to higher mean BAI ranks (minimal = 10.33, mild = 15.33, moderate = 21.46, severe = 31.30). At 3 months, the differences remained significant (χ^2^ (3) = 17.133, *p* = 0.001), with mean BAI ranks increasing across the severity levels (minimal = 9.92, mild = 15.92, moderate = 21.83, severe = 30.70). At 6 months, the trend persisted, showing significant differences (χ^2^ (3) = 19.823, *p* < 0.001) and a consistent increase in mean BAI ranks with higher BDI severity levels (minimal = 9.21, mild = 17.08, moderate = 21.33, severe = 32.20). These findings indicate that higher levels of depression (BDI) are associated with higher levels of anxiety (BAI) across all time points.

When analyzing BAI scores across the BDI severity groups at 3 months (χ^2^ (3) = 14.203, *p* = 0.003) and 6 months (χ^2^ (3) = 13.458, *p* = 0.004), significant differences were also observed. At 3 months, the mean BAI ranks increased from 10.94 in the minimal group to 15.92 in the mild group, 21.83 in the moderate group, and 30.70 in the severe group. At 6 months, the mean BAI ranks were lowest in the minimal group (10.00), increasing to 15.05 in the mild group, 25.33 in the moderate group, and 23.50 in the severe group. These results further support the association between higher depression severity (BDI) and higher anxiety levels (BAI) at different time points, reinforcing the relationship between these two variables.

The Mann–Whitney U test revealed significant differences in BAI scores between BDI severity groups across the three time points (diagnosis, 3 months, and 6 months). At diagnosis, significant differences were observed between groups with minimal depression (BDI 0–13) and severe depression (BDI 29–63) (U = 0.000, Z = −3.180, *p* = 0.001), as well as between groups with moderate depression (BDI 20–28) and severe depression (U = 8.000, Z = −2.320, *p* = 0.020). Similarly, significant differences were found between groups with mild depression (BDI 14–19) and severe depression (U = 0.500, Z = −2.659, *p* = 0.008). These results indicate that participants with higher levels of depression consistently exhibited higher levels of anxiety at diagnosis.

At 3 months, the trend persisted. Significant differences in BAI scores were observed between minimal and severe depression groups (U = 2.000, Z = −2.957, *p* = 0.003), moderate and severe depression groups (U = 9.000, Z = −2.220, *p* = 0.026), and mild and severe depression groups (U = 0.500, Z = −2.678, *p* = 0.007). These findings reinforce the association between greater depression severity and higher anxiety levels over time.

At 6 months, the analysis revealed significant differences in BAI scores between minimal and severe depression groups (U = 0.000, Z = −3.164, *p* = 0.002), moderate and severe depression groups (U = 4.000, Z = −2.754, *p* = 0.006), and mild and severe depression groups (U = 0.000, Z = −2.745, *p* = 0.006). These results confirm the consistent relationship between higher BDI severity levels and higher BAI scores across all time points, with significant differences observed between most severity groups.

The Mann–Whitney U test was conducted to compare BAI scores between BDI severity groups at 3 and 6 months. When comparing groups with minimal depression (BDI 0–13) and mild depression (BDI 14–19), no statistically significant differences were observed in BAI scores at 3 months or at 6 months. Similarly, no significant differences were found between groups with minimal and severe depression (BDI 29–63) at 3 months, although a slight trend toward significance was observed at 6 months (U = 2.500, Z = −2.037, *p* = 0.042).

When comparing groups with mild depression and moderate depression (BDI 20–28), significant differences were observed at both 3 months (U = 11.500, Z = −3.025, *p* = 0.002) and 6 months (U = 10.000, Z = −3.132, *p* = 0.002), with higher BAI scores in the moderate depression group. Similarly, significant differences were found between mild and severe depression groups at 3 months (U = 3.000, Z = −2.123, *p* = 0.034), but not at 6 months.

Finally, comparisons between moderate and severe depression groups showed no significant differences in BAI scores at either 3 months or 6 months. These findings suggest that while significant differences in anxiety levels exist between certain BDI severity groups, particularly between mild and moderate depression, the differences are less pronounced when comparing minimal and severe or moderate and severe depression groups at later time points.

## 4. Discussion

The findings of this study provide valuable insights into the psychological and sociodemographic characteristics of individuals with RRMS and their associations with anxiety and depression over a six-month follow-up period. These results align with and expand upon existing literature, highlighting the significant psychological burden associated with RRMS and the influence of sociodemographic and clinical variables.

Based on recent studies, the findings of this study align with existing literature on the prevalence and persistence of psychological symptoms in individuals with RRMS. Research has shown that anxiety and depression are common comorbidities in RRMS patients, with depression affecting up to 42.7% of individuals and generalized anxiety disorder affecting 26% of patients [51]. A systematic review and meta-analysis reported that anxiety affects approximately 21.4% of RRMS patients, while depression prevalence varies depending on disability levels and disease progression. Anxiety disorders have been reported in up to 35.6% of multiple sclerosis (MS) patients, further emphasizing the significant psychological burden of the disease [52].

Depression has been reported to have a lifetime prevalence of up to 50% in MS patients, with a 71% higher risk compared to the general population [53]. It is commonly associated with specific symptoms such as fatigue, irritability, and anhedonia [53,54]. Anxiety, on the other hand, has a prevalence of up to 35% in MS patients and is often linked to the emotional toll of diagnosis and disease progression [53].

Furthermore, significant associations were identified between higher levels of anxiety and depression and lower health-related quality of life (HRQoL) in RRMS patients, emphasizing the need for targeted psychological interventions [20,55].

From a nursing perspective, these findings underscore the critical role of nurses in addressing the psychological burden associated with RRMS. Nurses are uniquely positioned to perform early psychological screenings during routine clinical assessments, utilizing validated tools such as the GADS, BDI, and BAI to identify patients at risk of anxiety and depression.

These findings are consistent with the current study, which also identified sociodemographic and clinical factors, such as lower income and the presence of autoimmune diseases, as contributors to higher levels of anxiety and depression.

The sociodemographic profile of the study sample aligns with trends observed in previous research. Our sample consisted of 57.1% women and 42.9% men, which is consistent with the well-documented higher prevalence of MS among women. Studies have shown that women are approximately 1.7 to 3 times more likely to develop MS than men, particularly in the relapsing-remitting form of the disease [51]. Additionally, an increasing incidence of MS among women, particularly in late-onset cases, has been documented [56].

The mean age of participants in this study (38.29 years) also aligns with the typical age range of MS onset, which commonly occurs between the ages of 20 and 40 years. This finding is consistent with a study that reported an average age of onset at 30.3 years, with a range of 13 to 60 years [57]. Similarly, the high proportion of participants with a university degree (40%) reflects findings suggesting that MS tends to affect individuals with higher educational attainment, possibly due to the socioeconomic factors associated with healthcare access and diagnosis [55,58].

Employment status and income levels were found to have significant associations with anxiety and depression scores in this study. Specifically, participants with lower annual incomes (<€12,450) were more likely to be classified in the highest categories of anxiety and depression. This finding is supported by previous research, which has demonstrated that lower socioeconomic status (SES) is a significant risk factor for poor mental health outcomes in MS patients. For instance, individuals with lower SES experience significantly higher levels of anxiety, depression, and fatigue compared to those with higher SES [59].

Similarly, another study found that markers of lower SES, such as lower income and unemployment, were correlated with worse self-reported scores in depression and anxiety among MS patients [60]. Furthermore, unemployment and financial instability are among the strongest predictors of mental health challenges in MS patients, particularly in those with relapsing-remitting MS [59]. Lower income and unemployment have been strongly associated with higher rates of anxiety and depression in RRMS patients [51].

The clinical characteristics of the sample, including personal medical history and initial symptoms, also played a role in the psychological outcomes observed. For example, 17.1% of participants reported a history of autoimmune diseases, and these individuals were more likely to fall into the higher categories of anxiety and depression on the Beck scale. This finding aligns with research indicating that autoimmune diseases, such as multiple sclerosis, are strongly associated with elevated rates of depression and anxiety due to the chronic nature of the disease and its impact on quality of life [1]. The presence of comorbid autoimmune diseases further exacerbates the psychological burden in MS patients [55].

Furthermore, a history of mononucleosis was associated with higher depression scores, which is consistent with studies suggesting that Epstein–Barr virus (the cause of mononucleosis) may play a role in MS pathogenesis and influence disease progression and psychological outcomes. The link between Epstein–Barr virus infection and the onset of MS, as well as its potential role in exacerbating psychiatric symptoms such as depression and anxiety [51].

The most reported initial symptoms in our sample were hypoesthesia (31.4%), visual disturbances or paresthesia (20%), and diplopia (17.1%). These findings are in line with existing literature, which identifies sensory disturbances and visual impairments as common early symptoms of RRMS. Importantly, these symptoms can significantly impact quality of life and contribute to the development of anxiety and depression. Sensory and visual impairments are significantly associated with poorer mental health outcomes and increased emotional distress in MS patients [1,55].

The most used treatments in our sample were ocrelizumab (20%), cladribine (14.3%), and natalizumab (14.3%), which are all disease-modifying therapies (DMTs) widely used in RRMS management. These findings align with research highlighting the effectiveness of these treatments in reducing relapse activity. Ocrelizumab and natalizumab demonstrated superior efficacy in reducing relapses compared to cladribine, while all three treatments remain widely prescribed due to their disease-modifying properties [61]. While DMTs effectively reduce disease activity, they may not significantly mitigate psychological symptoms. In some cases, the burden of treatment, including side effects and the stress of long-term therapy, may contribute to emotional distress in MS patients [55,62].

Interestingly, our study found that students and employed participants were more likely to be classified in the highest anxiety categories on the Beck scale, while retirees and self-employed individuals were primarily distributed in the lower categories. This may reflect the additional stressors faced by individuals who are balancing work or studies with the challenges of managing a chronic illness. For example, employed MS patients frequently report higher anxiety levels due to workplace stress and concerns about disease progression affecting job performance [62]. Research has also shown that balancing employment with chronic illness management can exacerbate anxiety symptoms, especially among younger and middle-aged individuals [63].

One of the key findings of this study is the relative stability of anxiety and depression levels over the six-month follow-up period. On the GADS scale, 91.4% of participants were classified as “Probable” for anxiety at baseline, 3 months, and 6 months, with no significant changes in distribution. Similarly, depression scores on the GADS and BDI scales showed no statistically significant changes over time. These results suggest that anxiety and depression are persistent issues in RRMS patients, which is consistent with findings from other studies. It is important to note that, given the small sample size and the exploratory nature of the study, these results should be interpreted with caution, as they aim to identify potential trends rather than establish definitive conclusions. Anxiety and depression levels in MS patients have been reported to remain stable over time, even with treatment and supportive care [51,64]. Anxiety and depression are common and persistent among MS patients. A systematic review and meta-analysis found that 44.5% of MS patients experience some level of anxiety or depression, with a significant proportion reporting persistent symptoms over time [65].

This persistence of psychological symptoms in MS patients may be partly explained by shared neurobiological mechanisms. Fatigue, depression, and pain in MS share a common underlying mechanism involving alterations in the brain’s reward system, mediated by neuroinflammation and monoaminergic neurotransmission dysfunction. Neuroimaging studies have also shown functional connectivity alterations between the ventral striatum and the prefrontal cortex, which correlate with the severity of fatigue and depression symptoms [65]. Furthermore, elevated levels of neurofilament light chain (NF-L) in cerebrospinal fluid have been associated with axonal damage and disease activity in MS. Although no direct correlation has been found between NF-L levels and psychological symptoms in patients treated with fingolimod, anxiety and depression may still contribute to the onset of clinical relapses [66].

Elevated anxiety and depression symptoms in MS patients often remain resistant to treatment, further reinforcing their persistence over time [67].

The prevalence of anxiety and depression in our sample is higher than the rates reported in some studies but comparable to others. A study reported a prevalence of 26% for anxiety and 42.7% for depression in RRMS patients, which is slightly lower than the rates observed in our study. This discrepancy could be attributed to differences in sample characteristics, assessment tools, or cultural factors [51].

Similarly, a meta-analysis reported pooled prevalence rates of 30.5% for depression and 22.1% for anxiety across various MS subtypes, with slightly lower rates in RRMS compared to progressive forms of the disease. These findings align with the observation that depression and anxiety are highly prevalent in MS populations, though variability in diagnostic criteria and study designs may lead to differences in reported rates [65].

Furthermore, depression was the most reported psychiatric disorder among MS patients, with prevalence rates ranging from 37% to 54%, followed by anxiety disorders, which ranged from 14% to 41%. These findings are consistent with the high prevalence rates reported in our study and reinforce the importance of addressing these comorbidities in MS management [51].

Cultural and regional differences in the prevalence of mood disorders among MS patients highlight the importance of considering stigma surrounding mental health, as countries with higher stigma often report lower rates of anxiety and depression [52].

Additionally, our findings regarding the stability of anxiety and depression scores over time align with other studies, which found no significant changes in psychological symptoms over a one-year follow-up period. However, our study provides new insights into the specific symptoms of anxiety and depression that fluctuate over time, such as pessimism and loss of pleasure, which were not addressed in previous research [55].

The analysis of depressive symptoms in MS patients over time highlights the complexity of how specific symptoms evolve and persist. Research has consistently shown that depression is a common and significant comorbidity in MS, with its presentation often varying across different stages of the disease.

Non-pharmacological interventions have shown promise in addressing these persistent psychological symptoms. Cognitive-behavioral therapy (CBT), physical exercise, and patient education have been identified as effective strategies for reducing depressive symptoms in MS patients. Specifically, CBT demonstrated a standardized mean difference (SMD) of −4.04 (95% CrI = −6.80 to −1.45), while physical exercise (SMD = −3.62, 95% CrI = −6.55 to −0.85) and education (SMD = −2.94, 95% CrI = −5.69 to −0.25) also yielded significant improvements [68]. Home-based Pilates has further been associated with significant reductions in depression (QIDS: d = 0.70), anxiety (STAI: d = 0.30), and total fatigue (d = 0.76) in patients with mild-to-moderate MS [69].

As MS progresses and physical disability increases, depressive symptoms such as pessimism and loss of pleasure often worsen. This underscores the chronic nature of depressive symptoms and highlights the importance of monitoring specific aspects of depression, such as hopelessness and anhedonia, which may not consistently respond to treatment [1].

Depressive symptoms like sadness and guilt often remain stable over time, with a significant proportion of patients reporting mild to moderate levels of these symptoms throughout follow-up periods. However, severe symptoms, such as suicidal ideation, were less commonly reported, though they still warrant continuous monitoring due to their potential impact on patient safety [70].

Mindfulness-based cognitive therapy (MBCT) and cognitive rehabilitation therapy (CRT) have also been shown to improve depressive symptoms (d = −0.46) and mental HRQoL (d = −0.73 for MBCT; d = −0.45 for CRT) in MS patients [71]. These interventions provide additional therapeutic options for addressing the chronic psychological burden in MS.

A systematic review highlighted variability in depressive symptoms in MS patients, with some, such as feelings of worthlessness and guilt, remaining stable, while others, like fatigue and loss of interest, frequently worsen as MS progresses [27].

The analysis of individual BAI (Beck Anxiety Inventory) symptoms over time in MS patients provides valuable insights into the progression and persistence of anxiety-related symptoms. Anxiety is a common comorbidity in MS, with various studies highlighting its prevalence and diverse symptomatology.

Symptoms such as tingling and numbness are highly prevalent in MS patients and tend to worsen over time, particularly in those with higher levels of disease activity. This aligns with the observed progressive increase in higher scores for these symptoms in the BAI analysis, suggesting that physical symptoms of anxiety in MS may intensify as the disease progresses [52].

Conversely, symptoms like dizziness, lightheadedness, and feelings of heat are reported at low levels in MS patients, reflecting their lower prevalence compared to other anxiety-related symptoms such as nervousness or inability to relax [72].

Regarding severe symptoms, such as fear of the worst happening or fear of dying, these symptoms often decrease over time in MS patients, especially with adequate psychological support and treatment. This finding is consistent with the trend observed in the analysis, where higher scores for these symptoms decreased over the six-month period [60].

On the other hand, symptoms like nervousness and inability to relax are reported to be persistent in MS patients. These symptoms are among the most common and enduring manifestations of anxiety in MS, often requiring targeted interventions to improve long-term outcomes. Symptoms such as pallor and facial flushing are less commonly observed in MS patients with anxiety and are typically less prominent compared to other physical manifestations of anxiety [73].

The relationship between depression and anxiety in MS has been extensively studied in recent years, consistently highlighting their high prevalence and significant interplay. A strong correlation between these conditions (r > 0.8, *p* < 0.001) supports the notion that baseline levels of depression can significantly influence anxiety at subsequent time points, as shown in multiple studies [1,74]. Depression and anxiety are among the most common neuropsychiatric comorbidities in MS, frequently co-occurring due to shared biological mechanisms such as dysregulation of the hypothalamic–pituitary–adrenal (HPA) axis and inflammatory processes. These mechanisms not only exacerbate psychological distress but also contribute to poorer disease outcomes, including increased fatigue and reduced quality of life [1,27].

Furthermore, studies have shown that up to 50% of MS patients experience either depression or anxiety, with many individuals presenting both simultaneously. Patients with higher depression scores are significantly more likely to report elevated anxiety levels, reinforcing the bidirectional relationship between these conditions. This aligns with findings that baseline depression levels can predict future anxiety symptoms, emphasizing the importance of early detection and management [27,65]. Notably, effective treatment of depression at earlier stages could mitigate the progression or severity of anxiety over time, highlighting the need for integrated approaches to address these comorbidities [27].

Our findings revealed that specific symptoms of anxiety and depression fluctuated over time. For instance, symptoms such as pessimism and loss of pleasure showed a general trend toward worsening over the six months, while symptoms like sadness and guilt remained relatively stable. This highlights the importance of monitoring individual symptoms rather than solely relying on overall scores, as certain aspects of mental health may require targeted interventions.

Stress has also been identified as a key factor in MS, acting as a trigger for clinical and radiological relapses. During the COVID-19 pandemic, stress levels were significantly elevated, with a direct association observed between increased stress and a higher annualized relapse rate (ARR) in 2020 (*p* = 0.030) [75]. Additionally, sleep disorders are prevalent in MS patients, with studies showing that they experience significantly worse sleep quality compared to healthy controls (*p* < 0.001). Sleep quality is strongly correlated with levels of anxiety (r = 0.633, *p* < 0.001) and depression (r = 0.599, *p* < 0.001), highlighting the interconnected nature of these psychological symptoms [67]. Moreover, the atypical–melancholic mixed subtype is the most prevalent form of depression in MS patients, with an odds ratio (OR) of 2.22 (95% CI = 1.03–4.80) [76].

This persistence highlights the need for long-term psychological support led by nursing professionals, who can provide continuous monitoring and personalized care. Nurses are well-positioned to address the chronic nature of psychological comorbidities in MS, offering tailored interventions to improve coping mechanisms, reduce emotional distress, and enhance disease self-management.

Clinical recommendations emphasize the need to implement specific diagnostic strategies to differentiate major depressive disorder (MDD) symptoms from those of MS, particularly for the atypical–melancholic mixed subtype [76]. Additionally, prioritizing the treatment of depression and fatigue is essential for fostering cognitive reserve development in MS patients [77]. Comprehensive MS management should include psychological evaluations and support programs, especially during crises such as the COVID-19 pandemic [75,78]. Furthermore, therapeutic interventions should be personalized, addressing the specific symptoms and needs of MS patients to improve their quality of life and reduce the impact of psychological comorbidities [68,69].

These findings underscore the need for long-term psychological support and interventions tailored to address the chronic nature of mental health challenges in MS patients.

### 4.1. Implications for Clinical Practice

The findings of this study have several important implications for clinical practice. First, the persistence of anxiety and depression in RRMS patients highlights the need for routine psychological assessments as part of comprehensive MS care. Early identification and management of these symptoms are crucial to improving patients’ quality of life and overall well-being. Second, the associations between psychological symptoms and sociodemographic factors, such as income and employment status, underscore the importance of addressing social determinants of health in MS care. Providing financial and occupational support to patients could help mitigate some of the psychological burden associated with the disease [20,55].

Third, the findings suggest that while DMTs are effective in managing disease activity, they may not adequately address psychological symptoms. This highlights the need for integrated care models that combine pharmacological treatments with psychological and social interventions. For example, cognitive-behavioral therapy (CBT) and mindfulness have been shown to be effective in reducing anxiety and depression in MS patients [40,55].

Nurses can play a pivotal role in addressing these challenges by implementing nurse-led follow-up programs that monitor patients’ psychological well-being and provide timely interventions. For example, psychosocial interventions such as mindfulness-based therapies, CBT, and stress management programs have demonstrated effectiveness in improving mental health outcomes and health-related quality of life. By incorporating these strategies into routine nursing care, nurses can help mitigate the psychological burden of RRMS and promote better disease management.

### 4.2. Limitations and Future Research

While this study provides valuable insights, it is not without limitations. The small sample size (n = 35) limits the generalizability of the findings, and the lack of a control group makes it difficult to determine causality. Additionally, the reliance on self-reported measures of anxiety and depression may introduce bias, as participants may underreport or overreport their symptoms. The single-center design further limits the external validity of the findings, reducing their applicability to broader populations. Future research should aim to include larger, more diverse samples and consider using objective measures of psychological symptoms.

Another limitation is the relatively short follow-up period of six months. While this timeframe allowed for the identification of trends in anxiety and depression, longer follow-up studies are needed to better understand the long-term psychological impact of RRMS and its treatments. The potential influence of disease-modifying therapies (DMTs) on psychological outcomes must also be considered, as these treatments may exert both direct and indirect effects on mental health, potentially confounding the results. Future studies should explore the mechanisms underlying the associations between sociodemographic and clinical factors and psychological outcomes, as well as the potential role of interventions in mitigating these effects.

## 5. Conclusions

This study provides preliminary evidence of the psychological burden associated with RRMS, indicating that anxiety and depression persist over time despite treatment. Sociodemographic and clinical factors, such as lower income and the presence of autoimmune diseases, were identified as potential contributors to higher levels of anxiety and depression. However, given the small sample size and the exploratory nature of this pilot study, the findings should be interpreted with caution.

The results emphasize the need for integrated care models that address not only the physical but also the psychological and social aspects of MS. Further research with larger samples and longer follow-up periods is essential to validate these findings and to explore the predictive potential of the identified variables. Such research would contribute to the development of effective interventions aimed at improving the quality of life for individuals living with RRMS.

## Figures and Tables

**Table 1:** Spearman correlation between depression and anxiety level.

			Anxiety at Diagnosis	Depression at Diagnosis	Anxiety at 3 Months	Depression at 3 Months	Anxiety at 6 Months	Depression at 6 Months
Spearman Rho	Anxiety at diagnosis	Correlation coefficient	1.000	0.774 **	0.919 **	0.675 **	0.866 **	0.675 **
		Sig. (two-tailed)	.	0.001	0.001	0.001	0.001	0.001
		n	35	35	35	35	35	35
	Depression at diagnosis	Correlation coefficient		1.000	0.732 **	0.884 **	0.816 **	0.841 **
		Sig. (two-tailed)		.	0.001	0.001	0.001	0.001
		n		35	35	35	35	35
	Anxiety at 3 months	Correlation coefficient			1.000	0.692 **	0.831 **	0.580 **
		Sig. (two-tailed)			.	0.001	0.001	0.001
		n			35	35	35	35
	Depression at 3 months	Correlation coefficient				1.000	0.694 **	0.854 **
		Sig. (two-tailed)				.	0.001	0.001
		n				35	35	35
	Anxiety at 6 months	Correlation coefficient					1.000	0.738 **
		Sig. (two-tailed)					.	0.001
		n					35	35
	Depression at 6 months	Correlation coefficient						1.000
		Sig. (two-tailed)						.
		n						35

**. The correlation is significant at the 0.01 level (two-tailed).

## Data Availability

The datasets generated and/or analyzed during the current study are not publicly available but can be obtained from the corresponding author upon reasonable request, subject to privacy and ethical restrictions.

## Supplementary Materials

The following supporting information can be downloaded at https://www.mdpi.com/article/10.3390/nursrep16020039/s1. Table S1: Frequency and distribution of BDI symptoms at diagnosis and at 3 and 6 months; Table S2: Frequency and distribution of BAI symptoms at diagnosis and at 3 and 6 months.

## Public Involvement Statement

There was no public involvement in any part of this research.

## Guidelines and Standards Statement

This manuscript was drafted against the STROBE for Observational studies.
