# Supplementary material for: Psychological Burden in Relapsing-Remitting Multiple Sclerosis: Sociodemographic and Clinical Determinants of Persistent Anxiety and Depression over a Six-Month Follow-Up
Source: Nurs Rep. 2026 Jan 26;16(2):39. doi: 10.3390/nursrep16020039 (PMC12942824; doi:10.3390/nursrep16020039)
Supplement: Supplementary file 1 [file nursrep-16-00039-s001.zip › nursrep-4121598-supplementary.pdf]

Table S1. Frequency and distribution of BDI symptoms at diagnosis and at 3 and 6 months.

Item Number	Symptom	Diagnosis			3 months		6 months	
		Frequency	*n*	%	*n*	%	*n*	%
1	Pessimism	0	8	22.9	7	20	8	22.9
		1	15	42.9	20	57.1	16	45.7
		2	12	34.3	7	20	11	31.4
		3	0	0	1	2.9	0	0
2	Mood	0	7	20	9	25.7	9	25.7
		1	23	65.7	23	65.7	22	62.9
		2	4	11.4	3	8.6	4	11.4
		3	1	2.9	0	0	0	0
3	Failure	0	24	68.6	22	62.9	23	65.7
		1	7	20	12	34.3	10	28.6
		2	4	11.4	1	2.9	2	5.7
		3	0	0	0	0	0	0
4	Dissatisfaction	0	10	28.6	5	14.3	4	11.4
		1	15	429	23	65.7	22	62.9
		2	8	22.9	7	20	9	25.7
		3	2	5.7	0	0	0	0
5	Feelings of guilt	0	21	60	20	57.1	18	51.4
		1	12	34.3	12	34.3	12	34.3
		2	2	5.7	3	8.6	5	14.3
		3	0	0	0	0	0	0
6	Feelings of punishment	0	25	71.4	24	68.6	24	68.6
		1	9	25.7	9	25.7	9	25.7
		2	1	2.9	0	0	1	2.9
		3	0	0	2	5.7	1	2.9
7	Self-disapproval	0	12	34.3	10	28.6	9	25.7
		1	17	48.6	23	65.7	19	54.3
		2	5	14.3	2	5.7	6	17.1
		3	1	2.9	0	0	1	2.9
8	Self-criticism	0	15	42.9	15	42.9	7	20
		1	17	48.6	15	42.9	20	57.1
		2	3	8.6	4	11.4	8	22.9
		3	0	0	1	2.9	0	0
9	Suicidal thoughts or ideas	0	29	82.9	26	74.3	27	77.1
		1	5	14.3	9	25.7	8	22.9
		2	1	2.9	0	0	0	0
		3	0	0	0	0	0	0
10	Crying	0	7	20	8	22.9	9	25.7
		1	15	42.9	15	42.9	13	37.1
		2	12	34.3	10	28.6	10	28.6
		3	1	2.9	2	5.7	3	8.6
11	Fatigability	0	7	20	5	14.3	5	14.3
		1	15	42.9	18	51.4	17	48.6
		2	12	34.3	11	31.4	13	37.1
		3	1	2.9	1	2.9	0	0
12	Social withdrawal	0	15	42.9	8	22.9	5	14.3
		1	12	34.3	23	65.7	21	60
		2	8	22.9	4	11.4	9	25.7
		3	0	0	0	0	0	0
13	Indecisiveness	0	11	31.4	10	28.6	6	17.1
		1	17	48.6	18	51.4	22	62.9
		2	6	17.1	7	20	6	17.1
		3	1	2.9	0	0	1	2.9
14	Changes in physical appearance	0	15	42.9	12	34.3	15	42.9
		1	15	42.9	18	51.4	12	34.3
		2	5	14.3	5	14.3	8	22.9
		3	0	0	0	0	0	0
15	Loss of energy	0	3	8.6	4	11.4	2	5.7
		1	14	40	14	40	12	34.3
		2	15	42.9	16	45.7	21	60

		3	3	8.6	1	2.9	0	0
16	Changes in sleep patterns	0	7	20	7	20	7	20
		1	15	42.9	15	42.9	17	48.6
		2	11	31.4	13	37.1	11	31.4
		3	2	5.7	0	0	0	0
17	Irritability	0	10	28.6	8	22.9	6	17.1
		1	15	42.9	17	48.6	20	57.1
		2	10	28.6	10	28.6	9	25.7
		3	0	0	0	0	0	0
18	Loss of appetite	0	18	51.4	22	62.9	25	71.4
		1	11	31.4	6	17.1	6	17.1
		2	6	17.1	7	20	4	11.4
		3	0	0	0	0	0	0
19	Difficulty concentrating	0	7	20	7	20	5	14.3
		1	16	45.7	17	48.6	16	45.7
		2	10	28.6	10	28.6	13	37.1
		3	2	5.7	1	2.9	1	2.9
20	Fatigue or tiredness	0	6	17.1	3	8.6	3	8.6
		1	13	37.1	19	54.3	14	40
		2	11	31.4	12	34.3	17	48.6
		3	5	14.3	1	2.9	1	2.9
21	Loss of libido	0	10	28.6	11	31.4	11	31.4
		1	13	37.1	15	42.9	18	51.4
		0	11	31.4	8	22.9	5	14.3
		3	1	2.9	1	2.9	1	2.9

n=35

Table S2. Frequency and distribution of BAI symptoms at diagnosis and at 3 and 6 months.

Number of items	Symptom	Diagnosis			3 months		6 months	
		Frequency	n	%	n	%	n	%
1	Tingling or numbness	0	7	20	7	20	7	20
		1	9	25.7	10	28.6	8	22.9
		2	8	22.9	11	31.4	12	34.3
		3	11	31.4	7	20	8	22.9
2	Feeling of heat	0	15	42.9	16	45.7	15	42.9
		1	11	31.4	10	28.6	12	34.3
		2	6	17.1	6	17.1	6	17.1
		3	3	8.6	3	8.6	2	5.7
3	Leg tremors	0	23	65.7	19	54.3	21	60
		1	5	14.3	7	20	6	17.1
		2	6	17.1	7	20	5	14.3
		3	1	2.9	2	5.7	3	8.6
4	Inability to relax	0	8	22.9	6	17.1	5	14.3
		1	7	20	9	25.7	9	25.7
		2	10	28.6	8	22.9	10	28.6
		3	10	28.6	12	34.3	11	31.4
5	Fear of the worst happening	0	5	14.3	7	20	9	25.7
		1	8	22.9	9	25.7	7	20
		2	10	28.6	12	34.3	9	25.7
		3	12	34.3	7	20	10	28.6
6	Dizziness or lightheadedness	0	22	62.9	21	60	21	60
		1	9	25.7	12	34.3	10	28.6
		2	2	5.7	0	0	2	5.7
		3	2	5.7	2	5.7	2	5.7
7	Palpitations or tachycardia	0	22	62.9	18	51.4	22	62.9
		1	8	22.9	15	42.9	11	31.4
		2	5	14.3	1	2.9	1	2.9
		3	0	0	1	2.9	1	2.9
8	Feeling of instability or physical insecurity	0	7	20	3	8.6	5	14.3
		1	9	25.7	9	25.7	9	25.7
		2	9	25.7	14	40	12	34.3

		3	10	28.6	9	25.7	9	25.7
9	Terrifying thoughts	0	9	25.7	7	20	7	20
		1	6	17.1	8	22.9	11	31.4
		2	10	28.6	13	37.1	8	22.9
		3	10	28.6	7	20	9	25.7
10	Nervousness	0	2	5.7	2	5.7	1	2.9
		1	10	28.6	10	28.6	12	34.3
		2	7	20	14	40	12	34.3
		3	16	45.7	9	25.7	10	28.6
11	Feeling of suffocation	0	15	42.9	19	54.3	16	45.7
		1	9	25.7	7	20	12	34.3
		2	6	17.1	6	17.1	5	14.3
		3	5	14.3	3	8.6	2	5.7
12	Hand tremors	0	26	74.3	26	74.3	25	71.4
		1	4	11.4	4	11.4	4	11.4
		2	2	5.7	2	5.7	4	11.4
		3	3	8.6	3	8.6	2	5.7
13	Generalized trembling	0	10	28.6	11	31.4	8	22.9
		1	5	14.3	9	25.7	9	25.7
		2	8	22.9	5	14.3	15	42.9
		3	12	34.3	10	28.6	3	8.6
14	Fear of losing control	0	14	40	12	34.3	16	45.7
		1	10	28.6	9	25.7	9	25.7
		2	6	17.1	11	31.4	8	22.9
		3	5	14.3	3	8.6	2	5.7
15	Difficulty breathing	0	24	68.6	23	65.7	24	68.6
		1	4	11.4	7	20	4	11.4
		2	5	14.3	3	8.6	4	11.4
		3	2	5.7	2	5.7	3	8.6
16	Fear of dying	0	19	54.3	20	57.1	21	60
		1	4	11.4	9	25.7	8	22.9
		2	6	17.1	3	8.6	5	14.3
		3	6	17.1	3	8.6	1	2.9
17	Startling	0	9	25.7	6	17.1	4	11.4
		1	7	20	11	31.4	9	25.7
		2	8	22.9	13	37.1	17	48.6
		3	11	31.4	5	14.3	5	14.3
18	Digestive or abdominal discomfort	0	23	65.7	22	62.9	19	54.3
		1	8	22.9	9	25.7	10	28.6
		2	3	8.6	2	5.7	4	11.4
		3	1	2.9	2	5.7	2	5.7
19	Pallor	0	33	94.3	33	94.3	34	97.1
		1	2	5.7	2	5.7	1	2.9
20	Facial flushing	0	25	71.4	27	77.1	24	68.6
		1	5	14.3	3	8.6	6	17.1
		2	4	11.4	3	8.6	3	8.6
		3	1	2.9	2	5.7	2	5.7
21	Sweating (not due to heat)	0	19	54.3	19	54.3	18	51.4
		1	7	20	7	20	8	22.9
		0	7	20	7	20	7	20
		3	2	5.7	2	5.7	2	5.7

n=35
